# Drying methods for *Rheum tanguticum*: a comprehensive study of quality traits and metabolite dynamics

**DOI:** 10.3389/fphar.2026.1854690

**Published:** 2026-06-09

**Authors:** Zengrong Ye, Chen Chen, Shuo Zhao, Jianan Li, Jiaqi Jin, Tao Wang, Guoying Zhou

**Affiliations:** 1 School of Ecological and Environmental Engineering, Qinghai University, Xining, Qinghai, China; 2 Sichuan Zoige Alpine Wetland Ecosystem National Observation and Research Station, Southwest Minzu University, Chengdu, Sichuan, China; 3 College of Life Sciences, Huaibei Normal University, Huaibei, Anhui, China; 4 Northwest Institute of Plateau Biology, Chinese Academy of Sciences, Xining, Qinghai, China

**Keywords:** anthraquinones, differential metabolites, drying method, metabolic pathways, Rheum tanguticum, UPLC-MS/MS

## Abstract

Rheum tanguticum (*R. tanguticum*) originates from high-altitude regions such as Qinghai and Gansu in China. It serves as both a precious traditional medicinal botanical drug and a potential functional food, and its extensive pharmacological activities have stimulated global demand. However, existing research predominantly focuses on optimizing individual drying techniques or single bioactive metabolites, failing to integrate multi-omics technologies to elucidate the chemical and physical alterations induced by drying. To this end, we evaluated five drying methods by integrating an approach that links physical indicators with chemical composition, revealing that, for the preservation of physical indicators, especially color and rehydration properties, vacuum freeze drying (LD) is optimal; for the retention of the key pharmaceutical metabolite free anthraquinones, microwave drying (WB) is the most effective. Whereas in the comprehensive evaluation of multi-dimensional quality, SG is optimal. Metabolomic analysis confirmed that LD and YG were most effective in preserving key metabolites. Flavonoids, phenolic acids, and amino acids collectively form the core metabolites of differential metabolites in dried *R. tanguticum*, primarily enriched in pathways such as purine metabolism. Collectively, this work systematically elucidates the impact of drying on *R. tanguticum*’s metabolite profile and physicochemical traits, offering both a theoretical basis for its precision processing and a transferable framework for optimizing drying techniques in related medicinal edible plants.

## 1 Introduction

*Rheum tanguticum* (Maxim. ex Regel) Balf. (Polygonaceae) is a tall perennial botanical drug (Hu et al., 2022). It is native to China, where it has been utilized for millennia and is endemic to the Tibetan Plateau (Wang et al., 2012). In culinary applications, it is commonly used for making jam, brewing, and as a cooking ingredient (Lee et al., 2017); its tender rhizomes can also be consumed directly. In medicinal contexts, its mature rhizomes have long been valued as a traditional botanical drug (Zhao et al., 2024). Modern pharmacological analysis further reveals that it contains various active metabolites, such as anthraquinones (AQ) (Wang et al., 2022), which are associated with demonstrated therapeutic efficacy in the treatment of constipation, inflammation, and liver diseases (Huang et al., 2022; Wu et al., 2022). Nevertheless, the efficacy and quality of *R. tanguticum* in clinical applications are often constrained by drying processing methods. The drying process exerts a substantial influence on the appearance quality and chemical composition of the botanical drug (Zhong et al., 2024), which in turn modifies its metabolome and may consequently affect the final efficacy and safety.

Conventional drying techniques, including natural shade drying (YG) and solar drying (SG), though straightforward and accessible, frequently prove to be inefficient and are susceptible to the influence of environmental factors, resulting in variable botanical drug quality (Shen et al., 2022). Conversely, modern drying techniques, such as vacuum freeze-drying (LD) and microwave drying (WB), have emerged as a focal point in research due to their high efficiency, energy-saving and environmentally friendly characteristics (Yao et al., 2023). Despite the fact that these advanced drying techniques have been shown to better maintain the active ingredients and bioactivities of the botanical drugs, the dynamic perturbation mechanism of temperature gradient and changes in active substances on the metabolome of *R. tanguticum* during the drying process remains to be elucidated. This is especially true in the context of widely targeted metabolomics technology (a high-throughput targeted strategy based on known metabolite databases), particularly for *R. tanguticum*, for which a comprehensive analysis of the drying process, differential metabolites, and overall metabolic network dynamics is still lacking.

Evidence from metabolomics-driven investigations indicates that the drying protocol has a fundamental effect on the chemical signature of medicinal plants. For instance, Ge et al. (2025) found that sweating preserved essential oils in *Pogostemon cablin*, whereas oven drying enhanced antioxidants. Wang et al. (2025) identified over 920 differentially abundant metabolites (i.e., with altered levels induced by the treatments) in walnuts subjected to different drying treatments, linking secondary metabolite-nucleotide metabolic pathways to drying methods. Zhou et al. (2023) demonstrated that low-temperature drying can enhance the levels of tannins and ellagic acid in Kampo tea. Collectively, these studies suggest that there is a close relationship between drying parameters and the metabolic regulatory network that controls the accumulation of bioactive metabolites. In addition, a substantial body of research has confirmed that the drying process exerts a significant regulatory effect on the active metabolites of medicinal plants (Luo et al., 2025; Babaei Rad et al., 2025). However, the changes in metabolites and metabolic pathways of *R. tanguticum* induced by drying methods are relatively few, and the existing results are mostly focused on the analysis of a single constituent or a specific taxon. There is a paucity of studies revealing the response mechanism of drying methods from the perspective of the overall metabolic network. Furthermore, metabolomics, as an important branch of systems biology, has been widely utilized in the domains of plants, microorganisms, disease diagnosis and toxicology (Wang et al., 2010), with the capacity to systematically identify and quantitatively analyze all the metabolites of organisms, and to detect minor alterations in biological pathways to reveal the metabolic nature of the organism’s life activities (Sumner et al., 2003; Idle and Gonzalez, 2007; Wishart, 2019).

Therefore, this study aimed to systematically investigate the impact of different drying methods on the quality of *R. tanguticum*, by correlating changes in physicochemical properties with the dynamic variations in its metabolome. The study comprehensively analyzed and identified changes in specific active metabolites and the accumulation process of metabolites in *R. tanguticum* subjected to different drying methods, with a focus on elucidating the mechanisms underlying changes in metabolic products and pathways. The objective of this study is to elucidate the intrinsic relationship between drying processes, pharmacological activity, and metabolic pathways from multiple perspectives, thereby providing a theoretical foundation for the development of precise drying processes for *R. tanguticum*. Furthermore, it provides novel perspectives and methodologies for the research of drying processes for other botanical drugs.

## 2 Materials and methods

### 2.1 Materials

#### 2.1.1 Plant materials and pretreatment

Fresh *R. tanguticum* was harvested in July 2023 from the planting base of *R. tanguticum* in Donggou Township, Mutual Assistance County, Haidong City, Qinghai Province, China (102°7′18.1344″E, 36°53′38.166″N, altitude: 3070 m). The plants were artificially planted with 10-year-old *R. tanguticum* and were identified by Professor Guoying Zhou of Southwest Minzu University as *Rheum tanguticum* (Maxim. ex Regel) Balf. (Polygonaceae). A voucher specimen (No. HNWP00027186) has been deposited at the Herbarium of the Northwest Plateau Institute of Biology, Chinese Academy of Sciences (HNWP). After rinsing with purified water and air-dried to remove surface moisture, fresh *R. tanguticum* roots were transversely sliced into standardized 2-cm-thick segments at a position one-third from the root tip for subsequent drying treatments.

#### 2.1.2 Instruments and reagents

An Agilent-1260 HPLC high performance liquid chromatograph was equipped with a G1311C quadruple pump, a G1315D detector, a G1329B autosampler, and a G1316A column oven. The chromatographic columns were Agilent TC-C18 column (4.6 × 250 nm, 5 μm) and Agilent SB-C18 column (2.1 × 100 mm, 1.8 µm). Hot air drying was performed using a DHG-9425AE vertical blast drying oven, vacuum freeze drying was conducted with a FD-1A-50PLUS vacuum freeze dryer, microwave drying was carried out using an XRT-XG microwave drying.

Methanol and acetonitrile (HPLC grade, Shandong Yuwang & World New Materials); methanol, chloroform, NaHCO_3_, HCl, H_3_PO_4_, and formic acid (analytical grade; sources: Tianjin Damao, Sichuan Xilong, Sinopharm, Baiyin Liangyou, and Chengdu Cologne Chemical, respectively). Control aloe-emodin (110757–201607), rhein (110756–201913), emodin (110795–202011), chrysophanol (110796–201817), physcion (110758–201013), sennoside A (110824–201702), sennoside B (110825–201603), gallic acid (110831–200803) and catechin (110877–201604) were obtained from Shanghai Jizi Biochemical Technology Co.

### 2.2 Methods

#### 2.2.1 Drying method

Under uniform indoor temperature and humidity conditions, fresh *R. tanguticum* slices (2 cm thick) were dried until they reached a constant mass corresponding to the equilibrium moisture content for each drying condition (Argyropoulos et al., 2012), at which point drying was stopped and the slices were sealed and stored. For each drying condition, three biological replicates (n = 3, each from a different plant individual) were performed independently. The detailed experimental design is shown in the Supplementary Figure S4. The conditions were as follows: Hot air drying (HG): dried at 40 °C with a hot air flow rate of 2 m/s. LD: pre-frozen at -20 °C for 48 h, then transferred to a freeze dryer at -70 °C and 1000 Pa. WB: microwaved at 60 W and 80 °C with a rotation speed of 5 rpm. YG: dried in a shaded, well-ventilated area, turned twice daily. SG: solar-dried under direct sunlight during the day, and kept at -20 °C at night or during rainy weather to avoid moisture reabsorption.

#### 2.2.2 Analytical methods for bioactive metabolites

Control solutions were prepared by dissolving standards in appropriate solvents: Anthraquinone mixed standard (aloe-emodin, rhein, emodin, chrysophanol and physcion) at 0.22, 0.60, 0.17, 0.14, 0.30 mg/mL in methanol; Gallic acid and catechins at 0.40 and 1.60 mg/mL in methanol; Sennoside A and B at 0.40 and 0.80 mg/mL in 0.1% NaHCO_3_.

Test solutions were prepared as follows: Free anthraquinone (free AQ) were prepared with reference to the method of the Pharmacopoeia of the People’s Republic of China, 2025 edition (Chinese Pharmacopoeia Commission, 2025); Dianthrone glycosides (DAGs) and tannins were prepared with reference to the method of the previous researchers of the group (Wang et al., 2024).

HPLC conditions were as follows: Free AQs: Agilent TC-C18 (4.6 × 250 mm, 5 µm), methanol (A)/0.1% phosphoric acid (B), 85% A from 0 to 40 min, 1 mL/min, 25 °C, UV wavelength 254 nm, injection volume 10 µL. DAGs: Same column as above, methanol (A)/0.1% phosphoric acid (B), 0–12 min 42%–45% A, 12–15 min 45%–50% A, 1 mL/min, 40 °C, UV wavelength 340 nm, injection volume 10 µL. Tannins: Same column as above, acetonitrile (A)/0.1% formic acid (B), 5% A from 0 to 6 min, 5%–7% A from 6 to 7 min, 7%–15% A from 7 to 26 min, 1.0 mL/min, 26 °C, UV wavelength 280 nm, injection volume 20 µL. Each biological replicate was analyzed in triplicate (n = 3); data are mean ± SD.

The HPLC method established for the simultaneous quantification of nine characteristic metabolites, including aloe-emodin and rhein, was fully validated. All analytes demonstrated excellent linearity within their respective concentration ranges (R^2^ ≥ 0.9995). The method exhibited good precision, repeatability, and stability, with all relative standard deviation (RSD) values below 2.3%. Spike recovery experiments further confirmed the accuracy of the method, yielding average recoveries between 97.1% and 100.7%, with associated RSDs less than 2.1%. The detailed validation parameters are summarized in Supplementary Table S1 and Supplementary Figure S1.

#### 2.2.3 Widely targeted metabolomics analysis methods

Sample Preparation and Extraction: Briefly, the *R. tanguticum* powder was freeze-dried, ground, and extracted with cold 70% methanol containing an internal standard. After vortexing, centrifugation, and filtration through a 0.22 μm organic-solvent-compatible nylon membrane, the supernatant was subjected to UPLC-MS/MS analysis.

Chromatographic separation was performed on an Agilent SB-C18 column (2.1 × 100 mm, 1.8 µm). The mobile phase consisted of (A) ultrapure water containing 0.1% formic acid and (B) acetonitrile containing 0.1% formic acid. The gradient elution program was set as follows: 0.00–9.00 min, 5%–95% B; 9.00–10.00 min, maintained at 95% B; 10.00–11.10 min, B concentration decreased from 95% to 5% and equilibrated at 5% for 14 min. The flow rate was 0.35 mL/min, the column temperature was kept constant at 40 °C, and the injection volume was 2 µL.

Mass spectrometry analysis was carried out on a triple-quadrupole mass spectrometer operating in multiple reaction monitoring (MRM) mode. The key MS parameters were: ESI source temperature 500 °C; ion spray voltage ±5500 V; curtain gas 25 psi; ion source gases I and II at 50 and 60 psi, respectively; collision gas (nitrogen) set to medium. The analysis sequence was randomized, and no significant batch effect was detected based on QC sample monitoring (Figures 2a,b). For each metabolite, specific MRM transitions were monitored, with declustering potential (DP) and collision energy (CE) individually optimized. Metabolite identification and quantification was performed by putative matching of the accurate mass (Q1), MS/MS fragment spectra, and retention time (RT) against the self-built MetWare database (MWDB). The matching tolerances were set at 20 ppm for mass and 0.2 min for RT. Identification was validated only when a metabolite simultaneously met all tolerance criteria. Annotations were assigned confidence levels (Levels 1–3) based on spectral similarity and parameter consistency, following the reporting standards for metabolomics (Schymanski et al., 2014). Quantification was achieved by integrating the peak area of the characteristic fragment ion for each metabolite in MRM mode. Pooled QC samples were injected every 10 runs to monitor analytical reproducibility. Metabolites with CV > 0.5 in QC samples were excluded.

#### 2.2.4 Color difference determination method

Color differences before and after drying were measured using a color difference meter on five complete slices of *R. tanguticum* from each treatment group, both before and after drying. Each slice was measured three times and the average value was taken. During measurement, the sample completely covered the probe to eliminate scattered light. The L*, a* and b* values were recorded and the color difference after drying was calculated.

$$\Delta E={\left[{\left(L0\;*–L*\;\right)}^{2}+{\left(a0\;*–a*\;\right)}^{2}+{\left(b0\;*–b*\;\right)}^{2}\right]}^{\left(1/2\right)}$$ (1)

In the Equation 1, L_0_*, a_0_* and b_0_* represent the brightness, red-green and blue-yellow values before drying, and L*, a* and b* represent the values after drying. ΔE represents the final colour difference.

#### 2.2.5 Scanning electron microscopy methods

The microstructure of the examined samples was observed using a scanning electron microscope in the same position and under the same light conditions. The sample powder was placed on the scanning electron microscope stub and examined at an accelerating voltage of 2.0 kV.

#### 2.2.6 Method for determination of rehydration ratio (RR)

Dried samples (5.0 g) from each treatment were placed in a water bath at 60 °C with 300 mL of distilled water for 5 h. After removal, surface water was absorbed with filter paper, and the samples were weighed. The rehydration ratio was calculated using Equation 2:

$$RR=\frac{W_{b}}{W_{a}}$$ (2)

where $W_{b}$ is the weight after rehydration and $W_{a}$ is the weight after drying.

#### 2.2.7 Methods of leachate determination

Leachate content was determined by the hot infusion method, following General Rule 2201 in the Pharmacopoeia of the People’s Republic of China (2020 edition).

#### 2.2.8 Data statistical analysis methods

Multivariate statistical analysis can reduce dimensionality while retaining the original information, making it suitable for complex metabolomics data. In this study, Pearson correlation coefficients were calculated in R (version 4.1.2) to evaluate the consistency of the biological replicates. Unsupervised pattern recognition was then performed on all samples (including QC samples) using unit variance scaling and principal component analysis (PCA) to reveal overall metabolic differences and variability. Supervised OPLS-DA (MetaboAnalyst R 1.0.1, log_2_ transformation and centering) was then used for pairwise comparisons. Differential metabolites were screened based on VIP values. Finally, Metabolite identification was performed by matching accurate mass and retention time against our in-house standard metabolite library (MetMap database). Subsequently, the identified metabolites were annotated and subjected to pathway enrichment analysis using the Kyoto Encyclopedia of Genes and Genomes (KEGG) database.

All raw metabolomics data processing, including peak integration and correction across samples, was performed using the vendor’s software (Analyst 1.6.3 and MultiQuant). The processed data were then subjected to a t-test using Origin version 2021 software, with *P* < 0.05 considered statistically significant. Comprehensive evaluation was performed via entropy-weighted TOPSIS analysis using the SPSSAU platform and Microsoft Excel 2003.

## 3 Results and discussion

### 3.1 Physical properties analysis

#### 3.1.1 Colour and texture

The color parameters (L* and b*) served as critical indicators for evaluating the impact of drying methods on the visual quality of *R. tanguticum* roots (Önal et al., 2021). The fresh root sections exhibited significantly higher L (lightness) and b* (yellowness) values (Table 1), corresponding to a light yellow hue. All drying treatments markedly reduced these values. This substantial decrease in both L* and b* values suggests that drying processes may induce the degradation or transformation of light-colored metabolites or the conversion of pigment precursors (Rumicha et al., 2025), directly affecting the marketability and traditional appraisal of the botanical drug. The total color difference of *R. tanguticum* cross sections under different drying treatments was ranked as follows: LD < SG < HG < YG < WB. The L* and b* values of the cross sections under LD treatment were most similar to those of fresh *R. tanguticum*, and the total color difference was significantly lower than that of the other treatments. This finding indicated that after LD treatment, the color change in sections was minimal, enabling preservation of their original appearance, this is attributable to the vacuum anaerobic environment that inhibits enzymatic browning (Zeng et al., 2022), thereby minimizing material discoloration and structural collapse (Nowak and Jakubczyk, 2022). This outcome has been corroborated by numerous studies (Mi et al., 2024; Chao et al., 2023). The natural drying method resulted in a significantly smaller color difference for SG compared to YG. Both exhibited low L* values on the cross section, indicating a dark coloration, which appeared as a light reddish brown. Among the five methods, WB demonstrated the most pronounced color difference, with a discernible change in color before and after the drying process (Photographs comparing the appearance of samples before and after drying treatments can be found in Supplementary Figure S2).

**TABLE 1:** Colour and texture of *Rheum tanguticum* roots before and after different drying treatments.

Drying method	L^*^	a^*^	b^*^	△E^*^	Character
Fresh	42.63 ± 4.75	15.60 ± 2.35	32.21 ± 5.42	-	-
SG	23.21 ± 6.90b	10.36 ± 3.39ab	13.86 ± 3.10c	7.65 ± 3.97bc	Light, brittle
YG	21.10 ± 6.37b	8.10 ± 2.43b	14.83 ± 4.90c	12.97 ± 4.39ab	Firm, hard
WB	24.97 ± 6.25b	10.03 ± 2.71ab	16.86 ± 5.30c	14.75 ± 7.06a	Light, firm
LD	41.01 ± 4.50a	10.05 ± 1.82ab	25.52 ± 3.51a	6.61 ± 3.32c	Light, brittle
HG	23.95 ± 4.57b	8.22 ± 1.24b	16.73 ± 3.58c	8.77 ± 3.04bc	Firm, hard

L*: brightness value; a*: red-greenness value, b*: yellow-blueness value; △E*: total colour difference.

Different letters in the same column indicate significant differences between treatments (P < 0.05), X ± SD, n = 3.

As shown in Table 1, different drying strategies led to distinct moisture loss mechanisms, which regulated internal structural reorganization and ultimately determined texture density or looseness. In the process of LD, the moisture present in the material was first cooled to a temperature close to the freezing point. This was followed by the freezing of the moisture in a high vacuum environment, resulting in the conversion of the moisture to solid ice (Ratti, 2001). Subsequently, the ice crystals underwent direct sublimation from a solid to a gaseous state, without undergoing melting, within a high vacuum environment (Pei et al., 2014). The low-temperature drying process had been shown to result in a significant reduction in the moisture content of the material, with minimal changes in its physical and molecular structure. This had been demonstrated to create the light, brittle texture and ease of breakage that was characteristic of LD *R. tanguticum*. In the LD process, the moisture in the material is first cooled to a temperature close to the freezing point. Subsequently, the moisture is frozen in a high-vacuum environment, converting it into solid ice (Ratti, 2001). The ice crystals then undergo direct sublimation from solid to gas within a high-vacuum environment without melting (Pei et al., 2014). The low-temperature drying process has been shown to significantly reduce the moisture content of the material while causing minimal changes to its physical and molecular structure. This has been demonstrated to produce the light, brittle texture and friability characteristic of LD-treated *R. tanguticum*. YG is characterized by its natural environment, devoid of any external heat source. The material’s inherent moisture evaporates naturally into the ambient air. The internal cellular tissue gradually transforms into fibrous tissue, which confers greater stability on the dried material, resulting in a firm and hard texture. WB-treated *R. tanguticum* was characterized by a hard but light texture.

#### 3.1.2 Microstructure and rehydration ratio (RR)

**FIGURE 1:**
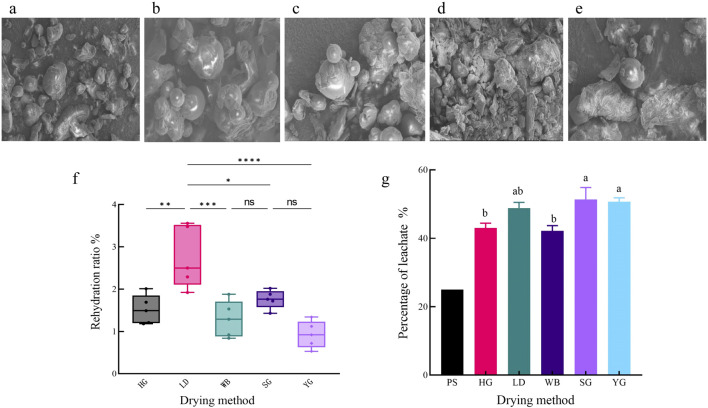
Graphs of physicochemical properties of *Rheum tanguticum* with different drying methods. Note: (**(a-e)** scanning electron microscope microstructural analyses, in which **(a)** HG, **(b)** YG, **(c)** SG, **(d)** WB, **(e)** LD; **(f)** graph of rehydration percentage share; **(g)** graph of leachate content) PS in Figure g is the Chinese Pharmacopoeia standard.

The microstructure of *R. tanguticum* under different drying methods is shown in Figure 1a-e (scanning electron microscopy images). The results indicated that the microstructure of *R. tanguticum* powder underwent significant changes depending on the drying method used. LD *R. tanguticum* powder particles exhibited a complete morphology (Xiao et al., 2024), a relatively smooth surface, relatively uniform particle size, and an absence of obvious crushed particle debris. Conversely, *R. tanguticum* powder subjected to HG at 40 °C exhibited a rougher and more uneven surface, with particles that were less uniform in size. In contrast, the morphology of *R. tanguticum* powder subjected to YG and SG was more similar, consisting of round particles of varying sizes. The agglomeration was dense, comprising both larger particles and smaller fragments. In comparison, the particles of WB-treated *R. tanguticum* powder were crumbly, densely distributed, and had a rough, irregular external texture, predominantly appearing as square-shaped blocks. In general, *R. tanguticum* samples processed by LD showed uniform particle distribution and orderly arrangement. This finding is consistent with previous research on the microstructure of *Cordyceps sinensis* (Zhao et al., 2025), which suggested that LD reduced damage to plant tissue structure (Mi et al., 2024; Ratseewo et al., 2025).

The rehydration ratio (RR), defined as the weight of rehydrated dried material relative to its dry weight, is an important index for evaluating the drying effect and water reabsorption capacity of botanical drugs (Jiang et al., 2022). The RR of *R. tanguticum* treated with different drying methods is presented in Figure 1f, and the rates were ranked from high to low as follows: LD > SG > HG > WB > YG. The RR of LD was significantly higher than that of the other drying methods, indicating that LD better preserved the internal cellular structure of *R. tanguticum* during drying, enabling the material to return to a near-original state more rapidly upon rehydration. This suggests that LD reduces damage to the cellular structure and creates a porous framework, thereby conferring the highest rehydration capacity (Jiang et al., 2014). The RR of YG was the lowest at 0.768 g, which is attributed to the long drying cycle at low temperature and in darkness; the slow moisture loss caused continuous shrinkage of the internal cellular structure, narrowing the water-outflow channels and ultimately reducing the RR. The SG temperature lay between LD and HG, and its RR was slightly higher than that of both.

#### 3.1.3 Leachate component

The leachate content is a significant indicator for evaluating the quality of botanical drugs, reflecting the amount of soluble substances present (He et al., 2024). The leachate content is directly related to the extraction of bioactive metabolites. The leachate content of *R. tanguticum* under different drying treatments was significantly higher than the 25% standard stipulatedin the Chinese Pharmacopoeia (Figure 1g), with an average of 46.0%. Further analysis of the data revealed that the proportion of leachate content was higher in SG and YG treated *R. tanguticum*. The higher leachate content in these groups suggests a greater yield of soluble extracts under these drying conditions. This may imply a better potential for the extraction of bioactive metabolites, although the specific composition and pharmacological activity of these soluble substances require further verification.

### 3.2 Bioactive metabolites analysis

#### 3.2.1 Free anthraquinone

Within *R. tanguticum*, anthraquinones are considered core pharmacodynamic metabolites (Zhao et al., 2023). Their free and bound forms have been reported to exert synergistic laxative, antibacterial, and anti-inflammatory effects (Bao et al., 2024). In the present study, we analyzed the free anthraquinone (free AQ) content of *R. tanguticum* processed by different drying methods (Table 2). As shown in Table 2, different drying methods significantly affected the free AQ content of *R. tanguticum* (*P* < 0.05), and the free AQ content ranged from 0.68% to 5.30%, with a descending order of WB > YG > SG > HG > LD. The free AQ content of *R. tanguticum* from all five treatment methods was higher than the Chinese Pharmacopoeia standard (0.20%). No significant difference was observed between SG and HG treatments, whereas significant differences were found among WB, YG, and LD treatments (*P* < 0.05). Compared to SG, YG retained better free AQ content, especially for rhein, chrysophanol, and physcion. Four free AQs (aloe-emodin, emodin, chrysophanol, and physcion) were significantly more abundant in the WB treatment than in the other modalities. LD showed the lowest free AQ content among the five treatments for most of the detected compounds. This finding is consistent with a recent study on rhubarb, which demonstrated that freeze-drying reduces anthraquinone content in rhubarb under certain conditions (Cheng et al., 2025).

**TABLE 2:** Free AQ content of *Rheum tanguticum* under different drying methods.

Drying method	Bioactive metabolites (%, n = 3)					
	Aloe-emodin	Rhein	Emodin	Chrysophanol	Physcion	Total
HG	0.067 ± 0.003b	1.319 ± 00.069c	0.020 ± 0.001b	0.246 ± 0.015c	0.076 ± 0.002c	1.727 ± 0.086c
LD	0.033 ± 0.001c	0.495 ± 0.022d	0.009 ± 0.001b	0.105 ± 0.002d	0.036 ± 0.001d	0.677 ± 0.025d
WB	0.289 ± 0.031a	3.988 ± 0.427a	0.068 ± 0.027a	0.696 ± 0.081a	0.259 ± 0.028a	5.300 ± 0.540a
SG	0.061 ± 0.002b	1.339 ± 0.025c	0.021 ± 0.001b	0.264 ± 0.007c	0.101 ± 0.004c	1.785 ± 0.038c
YG	0.067 ± 0.001b	1.900 ± 0.065b	0.025 ± 0.001b	0.313 ± 0.017b	0.124 ± 0.006b	2.428 ± 0.090b

In the table, the letters a‐d indicate significant statistical differences between groups. Data that do not share a common letter differ significantly from each other (p < 0.05).

#### 3.2.2 Dianthrone glycoside

The contents of dianthrone glycosides (DAGs, primarily sennoside A and B) in *R. tanguticum* under different drying methods are presented in Table 3. Drying treatments significantly affected DAG content (*P* < 0.05). The LD and YG treatments yielded significantly higher DAG content than the other methods, with no significant difference between them. This enhanced retention under low-temperature (LD) and shaded, slow-dehydration (YG) conditions is consistent with reports that such environments minimize thermal degradation and photolytic cleavage of labile glycosidic bonds in DAGs (Goppel and Franz, 2004; Meier et al., 2024). In contrast, the WB treatment yielded the lowest total DAG content (3.83%), which can be attributed to localized overheating induced by endogenous microwave heating, leading to degradation of thermolabile sennosides (Nukasani et al., 2017). Among natural drying methods, YG (6.10%) surpassed SG (5.52%), further supporting the protective role of shading against photo-degradation (Meier et al., 2024). Similarly, a study on Cassia angustifolia found that shade drying retained higher sennoside content compared to other drying methods (Nilofer et al., 2021). Therefore, to optimize DAG retention, the LD or YG process is recommended.

**TABLE 3:** Content of dianthrone glycosides and tannins in *Rheum tanguticum* under different drying methods.

Drying method	Bioactive metabolites (%, n = 3)					
	Sennoside a	Sennoside B	Dianthrone glycosides total	Gallic acid	Catechin	Tannins total
HG	3.324 ± 0.126b	1.419 ± 0.060b	4.744 ± 0.185b	0.339 ± 0.001b	3.525 ± 0.276d	3.865 ± 0.276c
LD	4.363 ± 0.173a	1.784 ± 0.065a	6.147 ± 0.238a	0.150 ± 0.001b	9.475 ± 0.037a	9.625 ± 0.036a
WB	2.760 ± 0.018c	1.069 ± 0.009c	3.829 ± 0.028c	1.582 ± 0.108a	6.916 ± 0.081b	8.498 ± 0.189a
SG	3.887 ± 0.004a	1.631 ± 0.002a	5.518 ± 0.002a	0.206 ± 0.008b	4.215 ± 0.135d	4.421 ± 0.128c
YG	4.243 ± 0.107a	1.855 ± 0.038a	6.098 ± 0.144a	0.227 ± 0.008b	5.615 ± 0.163c	5.842 ± 0.171b

In the table, the letters a‐d indicate significant statistical differences between groups. Data that do not share a common letter differ significantly from each other (p < 0.05).

#### 3.2.3 Tannins

The tannin content in *R. tanguticum* was significantly influenced by the drying method (*P* < 0.05, Table 3). The highest total tannin content was achieved by LD. This is attributed to the combined effect of low temperature and an oxygen-limited environment in LD, which effectively suppresses polyphenol oxidase activity and non-enzymatic oxidation, thereby preserving phenolic metabolites (Brito et al., 2002). Among traditional methods, YG was superior to SG in tannin retention, consistent with the known protective effect of avoiding direct solar radiation against the photo-oxidation of polyphenols (Jiang et al., 2024). Notably, a compositional shift was observed under LD conditions: gallic acid content was relatively low (1.50 mg/g), whereas catechin was prominently preserved (94.75 mg/g). This suggests that the low-oxygen environment may inhibit the oxidative conversion of precursor substances (e.g., ellagic acid) or differentially affect the stability of specific phenolic acid derivatives (De Beer et al., 2023).

### 3.3 Metabolomics analysis

To elucidate the mechanisms underlying quality variations in *R. tanguticum* across five drying processes, small-molecule metabolites in SG, YG, WB, LD and HG samples were identified and quantified through metabolomic analysis.

#### 3.3.1 Multivariate statistical analysis of metabolites

**FIGURE 2:**
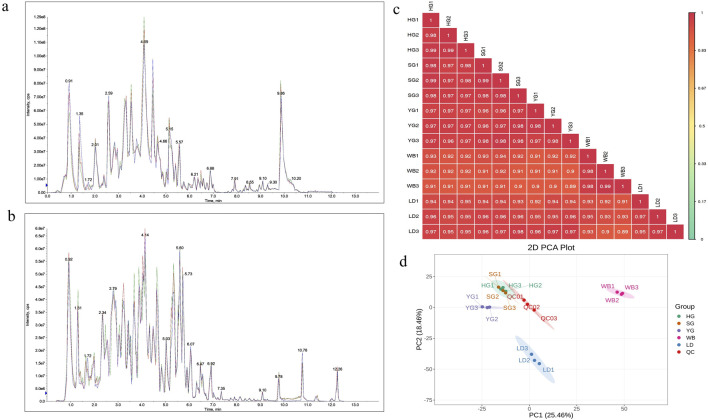
Multivariate statistical analysis of metabolites. Note: **(a)** overlap of TIC for positive ion mode samples; **(b)** overlap of TIC for negative ion mode samples; **(c)** correlation coefficients between samples; **(d)** two dimensional planar plot of PCA scores.

To assess the effect of different treatments on the metabolome of *R. tanguticum*, five drying modes (HG, SG, YG, WB and LD) were used. Analyses were carried out using widely targeted metabolomics techniques. The results, including the total ion current (TIC) curves, are shown in Figures 2a,b; the TIC curves for metabolite detection in positive and negative ion modes for the QC samples showed high overlap, i.e., the retention times and peak intensities were consistent, indicating that the mass spectrometry had good signal stability and that the data for the same samples detected at different times were reliable. Repeated correlation analysis using Pearson’s correlation coefficient (Figure 2c) demonstrated that the correlation coefficients of the samples within each group were above 0.95; this suggested that the intra-group correlations were high relative to inter-group correlations, and that the obtained differential metabolites were reliable. Further analysis via PCA (Figure 2d) revealed that the three replicate samples within each group clustered together in the PCA plot, whereas the YG, LD and WB groups remained distinct from one another. This indicated significant differences in metabolites among these three drying methods. Metabolite distributions for SG- and HG-treated samples exhibited relative clustering, suggesting similarities in their metabolite compositions. PC1, PC2 and PC3 collectively explained approximately 54% of the total variance, reflecting the multi-dimensional complexity of the metabolic response to different drying methods.

#### 3.3.2 Metabolite profile characterisation

**FIGURE 3:**
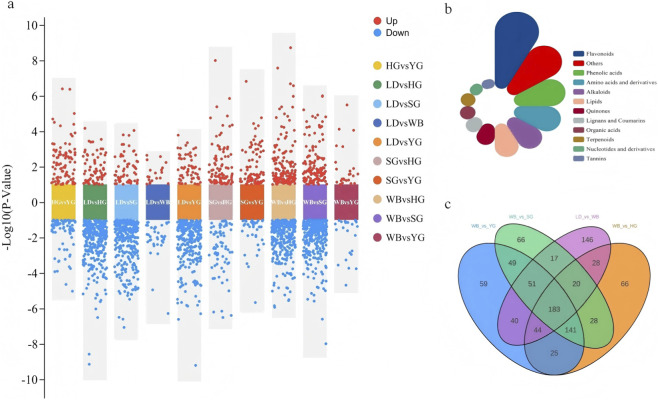
Characterisation of metabolite profiles of *Rheum tanguticum* with different drying methods. Note: **(a)** differential metabolite volcano plot; **(b)** metabolite classification chart; **(c)** metabolite species interaction comparison pistil diagr.

A total of 1096 significantly altered metabolites (screened by criteria: VIP > 1.0, FDR-adjusted *P* < 0.05, |log_2_FC| ≥ 1) across 12 categories were identified in the HG, SG, YG, WB and LD samples (Figure 3b). The most diverse categories were flavonoids (223 compounds), phenolic acids (134 compounds) and lipids (121 compounds), as classified by the in-house MetWare Database (MWDB) system. Flavonoids were not only abundant in *R. tanguticum* but have also been reported in rosehips (Zhou et al., 2024) and *Eucommia ulmoides* (Hong et al., 2025; Du et al., 2022). They have been shown to possess significant antioxidant properties (Fan et al., 2025) and to be involved in cancer prevention (Pyo et al., 2024) and liver protection (Chen et al., 2024). Furthermore, secondary metabolites rich in quinones, tannins, phenolic acids, flavonoids and organic acids were identified, whereas lipids, nucleotides and derivatives, and terpenoids were less abundant (Figure 3b). These findings suggest that these metabolites may be more sensitive to drying conditions (Sokač et al., 2022; Babaei Rad et al., 2025).

**FIGURE 4:**
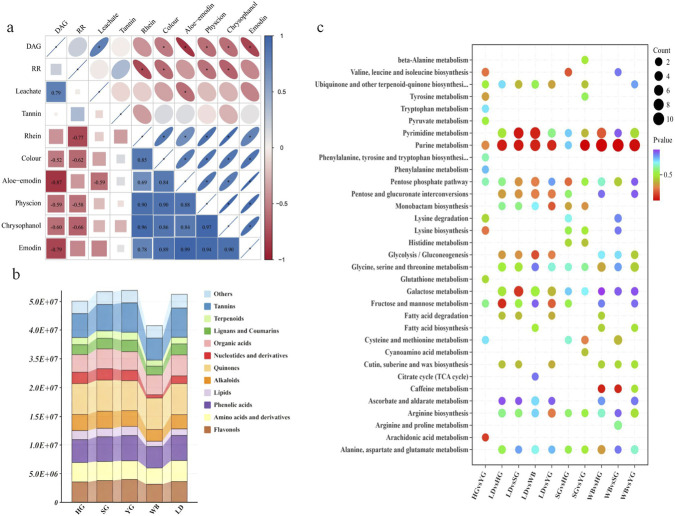
**(a)** multiple indicator correlation analysis; **(b)** metabolite content comparison chart; **(c)** differential metabolite comparisons between groups KEGG enrichment plot.

The orthogonal partial least squares-discriminant analysis (OPLS-DA) models exhibited good separation and predictability (e.g., for SG vs. HG: R^2^Y = 1, Q^2^ = 0.98; see Supplementary Figure S3). To elucidate the differential expression of metabolites in *R. tanguticum* samples subjected to different drying processes, we calculated the concentrations of twelve metabolite classes obtained using five different drying methods, as shown in Figure 4b. A comprehensive analysis of abundance levels across these twelve classes revealed significant differences in metabolite content among the *R. tanguticum* groups. Specifically, the HG, SG and YG groups exhibited higher flavonoid content than the WB and LD groups, while the WB and LD groups showed lower nucleotide and derivative abundance (Figure 4b). The SG group contained approximately 1.46 times more nucleotides and derivatives than the LD group, while the LD and YG groups exhibited 33.94% and 34.35% higher tannin levels than the WB group, respectively. Amino acids and their derivatives were most abundant in LD and least abundant in WB, with a 28% difference between these two groups. Regarding phenolic acids, the WB group showed reductions of 18% and 12% compared to the YG and HG groups, respectively. These findings indicate that drying processes significantly impact the levels of flavonoids, nucleotides and derivatives, tannins, and amino acids and their derivatives in *R. tanguticum*.

**FIGURE 5:**
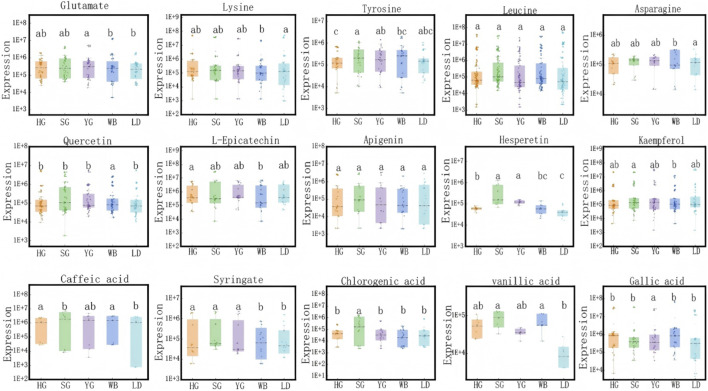
Comparison chart of primary monomer content.

Subsequent analysis of representative monomeric metabolites demonstrated that drying methods had a significant impact on the levels of key bioactive substances (Tang et al., 2025; Bashir et al., 2025) (Figure 5). This study showed that the vacuum-hypoxic environment (LD) significantly increased the content of the amino acids lysine and asparagine. Lysine, a cornerstone of protein synthesis, is crucial for childhood growth and development (Gunarathne et al., 2025), while asparagine exerts metabolic regulatory functions by accelerating hepatic detoxification and alleviating fatigue (Wu et al., 2015). Significant enrichment of tyrosine, kaempferol and chlorogenic acid was observed in the SG sample, indicating potential antioxidant, organ-protective and metabolic-regulatory effects (Bangar et al., 2023). The YG sample exhibited substantial elevation of glutamic acid and gallic acid. In contrast, WB promoted the accumulation of quercetin and vanillic acid, while HG, at elevated temperatures, only increased epicatechin. These differences indicate that drying methods substantially influence the metabolite composition and functional metabolite abundance of *R. tanguticum*. Such variations may arise from differing drying factors, such as processing duration or temperature-humidity conditions (Ryu et al., 2025).

#### 3.3.3 Differential metabolites and KEGG pathway

In order to comprehensively analyze the regulatory mechanisms of different drying methods on *R. tanguticum* metabolites, the fold change and statistical significance of metabolites were presented in volcano plots (Figure 3a). This approach facilitated more intuitive screening and presentation of significantly differentially expressed metabolites. A total of 170 significant differential metabolites were annotated and putatively identified between HG and YG (91 up-regulated and 79 downregulated), 378 between LD and HG (70 upregulated and 308 downregulated), 368 between LD and SG (57 up-regulated and 311 down-regulated), and 441 between LD and WB (176 upregulated, 265 downregulated). Between LD and YG, 59 up-regulated and 285 down-regulated differential metabolites were identified. Between SG and HG, 99 were up-regulated and 71 down-regulated. Between SG and YG, 88 were upregulated and 39 downregulated. Between WB and HG, 455 significant differential metabolites were identified (153 up-regulated, 302 downregulated); between WB and SG, 472 (127 up-regulated, 345 down-regulated); and between WB and YG, 537 (178 up-regulated, 359 downregulated).

The identification of key metabolites proved instrumental in the analysis of *R. tanguticum* metabolites from different drying methods (Xing et al., 2025). The volcano plots (Figure 3a) were used to select the four comparison groups that exhibited the most significant variation in the number of differential metabolites, i.e., WB vs. YG, WB vs. SG, WB vs. LD, and WB vs. HG. These groups were then plotted as a petal diagram, as shown in Figure 3c, to illustrate the common and unique metabolites across the various drying comparison groups. The four comparison groups contained 59, 66, 146, and 66 unique differential metabolites, respectively, and a total of 183 common metabolites were identified among these groups.

KEGG pathway enrichment analysis was employed to elucidate the signature metabolic or physiological processes involved in *R. tanguticum* under different drying methods (Luo et al., 2025). The enrichment significance was evaluated by p-value (<0.05) and Rich Factor. The findings demonstrated that the purine metabolism pathway exhibited significant enrichment across all eight comparison groups (Figure 4c), with DAMs being particularly abundant under WB conditions relative to SG, YG, and LD. This suggests that the purine metabolism pathway was most significantly affected under the WB drying condition. Conversely, pathways including beta-alanine metabolism, tryptophan metabolism, pyruvate metabolism, phenylalanine, tyrosine and tryptophan biosynthesis, phenylalanine metabolism, glutathione metabolism, citrate cycle (TCA cycle), and arachidonic acid metabolism appeared in only one comparison group each. Under LD conditions, metabolites associated with pyrimidine metabolism and pentose and glucuronate pathways showed more extensive changes. HG exhibited higher levels of enrichment in amino acid metabolism (valine, leucine and isoleucine biosynthesis). Furthermore, ubiquinone and other terpenoid-quinone biosynthesis, as well as the pentose phosphate pathway, were observed in the YG comparison group. The integration of KEGG enrichment metabolic pathways for differentially expressed metabolites among *R. tanguticum* groups under five drying conditions (Figure 4c) enabled the identification of pathways such as purine metabolism, pyrimidine metabolism, and galactose metabolism as being highly enriched. This finding is similar to a metabolomic study on *Chrysanthemi Flos*, which reported that the differentially accumulated metabolites were mainly enriched in purine metabolism, pyrimidine metabolism, and other metabolic pathways (Zhang et al., 2024). Across the 10 comparison groups, a total of 105 metabolic pathways were implicated. These additionally encompassed: purine metabolism, pyrimidine metabolism, galactose metabolism, fructose and mannose metabolism, pentose and glucuronate interconversions, pentose phosphate pathway, glycolysis/gluconeogenesis, arginine biosynthesis, monobactam biosynthesis, glycine, serine and threonine metabolism, alanine, aspartate and glutamate metabolism, cutin, suberine and wax biosynthesis, ubiquinone and other terpenoid-quinone biosynthesis, ascorbate and aldarate metabolism, caffeine metabolism, fatty acid degradation, cysteine and methionine metabolism, lysine biosynthesis, valine, leucine and isoleucine biosynthesis, and fatty acid biosynthesis. The enrichment of purine metabolism may be closely correlated with the color, flavor, and bioactive metabolites of *R. tanguticum*, and these metabolites are key substances that enhance food palatability (Sun et al., 2020). Pyrimidines, being structural components of nucleic acids (DNA and RNA), were abundant in nucleic acid-rich food that provides exogenous pyrimidines to the human body (Garavito et al., 2015).

### 3.4 Correlation analysis

After separately listing the five free AQ contents, we performed a correlation analysis on color, rehydration ratio (RR), leachate, and other bioactive metabolites. The results revealed multiple highly significant positive and negative correlations (|r| > 0.70, *P* < 0.01) among these metabolites (Figure 4a). However, the correlations among the three physical indicators (color, RR, leachate) were less pronounced. Specifically, color exhibited significant positive correlations with all five free AQ indicators: chrysophanol (r = 0.86), emodin (r = 0.89), and physcion (r = 0.90). RR showed a negative correlation with rhein (r = –0.77), while leachate demonstrated a positive correlation with dianthrone glycosides (DAG) (r = 0.79), suggesting a potential association, although the underlying mechanism remains to be elucidated. Among the five free AQ indicators, emodin exhibited a near-perfect positive correlation with aloe-emodin (r = 0.99), whereas its correlation with chrysophanol (r = 0.88) was slightly lower but still statistically significant. Furthermore, a strong negative correlation between free AQs and DAG was observed (r = –0.89). In summary, the correlation matrix (Figure 4a) suggests that there may be complex synergistic and antagonistic relationships between the physical and chemical constituents in dried *R. tanguticum*, providing correlational clues for variations in its metabolic products.

### 3.5 Comprehensive evaluation analysis

The entropy-weighted TOPSIS method was employed to conduct a comprehensive analysis of standardized physical characteristic indicators (color, RR value, and leachate) and chemical composition indicators (free AQ, DAG, and tannin) of *R. tanguticum* (Table 4). The selection of these indicators is based on their clear pharmacological relevance, with free AQs having anti-inflammatory and hepatoprotective activities, DAGs being used for laxative effects, and tannins exhibiting astringent, hemostatic, and antidiarrheal properties. All three are recorded in the Chinese Pharmacopoeia. In addition, physical attributes such as color, RR, and leachate are also important indicators for evaluating processing quality. The multi-criteria evaluation revealed the following hierarchy: SG > WB > YG > LD > HG. Specifically, SG demonstrated outstanding performance across multiple key indicators, particularly achieving high scores in the physical indicators of leachate and color, while maintaining mid-level performance in chemical indicators, resulting in the highest comprehensive evaluation ranking. WB secured the second position due to its significant advantage in retaining free AQ. YG exhibited performance comparable to SG in several key indicators, with high scores in DAG and leachate, though it showed considerable deviation in color. LD achieved the highest scores in RR, color, and tannin. In the comprehensive evaluation, HG ranked lower in most indicators, ultimately placing fifth.

**TABLE 4:** Comprehensive quality evaluation results of *Rheum tanguticum* under different drying methods.

Drying method	*d* ^+^	*d* ^-^	*c*_*i*_	Sequence
SG	0.1446	0.1807	0.5555	1
YG	0.1811	0.1807	0.4994	3
WB	0.1929	0.1964	0.5045	2
LD	0.2054	0.1958	0.4880	4
HG	0.1806	0.1468	0.4484	5

It should be noted that the five drying methods differ in multiple parameters, including temperature, time, and oxygen exposure. Therefore, the observed differences cannot be attributed to any single factor. This study aims to evaluate the overall performance of different drying techniques for practical application rather than to isolate the causal effects of individual variables. This limitation should be taken into consideration when interpreting the ranking. These correlation and pathway results are hypothesis-generating and require further experimental validation.

## 4 Conclusion

This study systematically evaluated the effects of five drying methods on the quality of *R. tanguticum* by integrating multiple analytical techniques. Key findings demonstrated that LD provided optimal color and rehydration properties, YG yielded the highest total content of dianthrone glycosides, while WB promoted the conversion of free AQ. Modern drying methods (LD, WB) were superior to traditional techniques in retaining tannins and DAGs. Metabolite profiling identified flavonoids, phenolic acids, and amino acids as key metabolites affected by drying, with purine metabolism notably altered under WB. Correlation analysis revealed a strong positive relationship between physical properties and chemical composition, especially between color and free anthraquinones. In practice, the optimal drying method depends on the specific application: LD for sensory quality, WB for key pharmaceutical metabolites, and SG for overall balanced quality. These results provide a scientific basis for precision drying of *R. tanguticum* and offer valuable insights for quality control in other rhizome-based Chinese botanical drugs.

## Data Availability

The original contributions presented in the study are included in the article/Supplementary Material, further inquiries can be directed to the corresponding author.

## Supplementary material

SUPPLEMENTARY FIGURE S1 HPLC of bioactive metabolites: test sample and reference std. Note: **(a,b)** Anthraquinone mixture reference standard (left) and test sample (right); **(c,d)** Tannin mixture reference standard (left) and test sample (right); **(e,f)** DAG mixture reference standard (left) and test sample (right). **(b)** 1. Aloe-emodin; 2. Rhein; 3. Emodin; 4. Chrysophanol; 5. Chrysophanol; **(d)** 1. Gallic acid; 2. Catechin; **(f)** 1. Sennoside B; 2. Sennoside A.

SUPPLEMENTARY FIGURE S2 R. *tanguticum* before and after drying comparison.

SUPPLEMENTARY FIGURE S3 Permutation plots for all OPLS-DA models Note: **(a)** LD vs. YG, **(b)** HG vs. YG, **(c)** LD vs. WB, **(d)** LD vs. HG, **(e)** LD vs. SG, **(f)** SG vs. YG, **(g)** SG vs. HG, **(h)** WB vs. SG, **(i)** WB vs. HG, **(j)** WB vs. YG.

SUPPLEMENTARY FIGURE S4 Experiment Design Diagram.

SUPPLEMENTARY FIGURE S5 PC1 control chart of QC and test samples across injection order.
