# Supplementary figures and images for: Drying methods for Rheum tanguticum: a comprehensive study of quality traits and metabolite dynamics
Source: Front Pharmacol. 2026 Jun 9;17:1854690. doi: 10.3389/fphar.2026.1854690 (PMC13287020; doi:10.3389/fphar.2026.1854690)

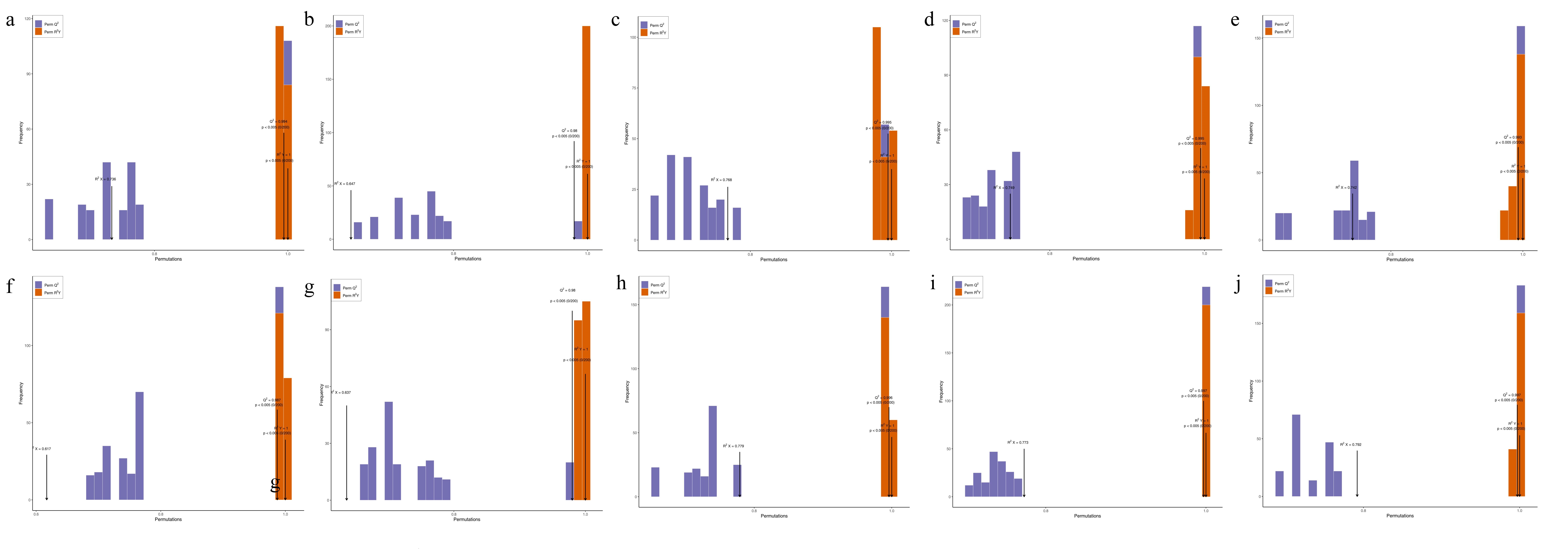

Supplement: Supplementary file 1 [file Image3.jpg]

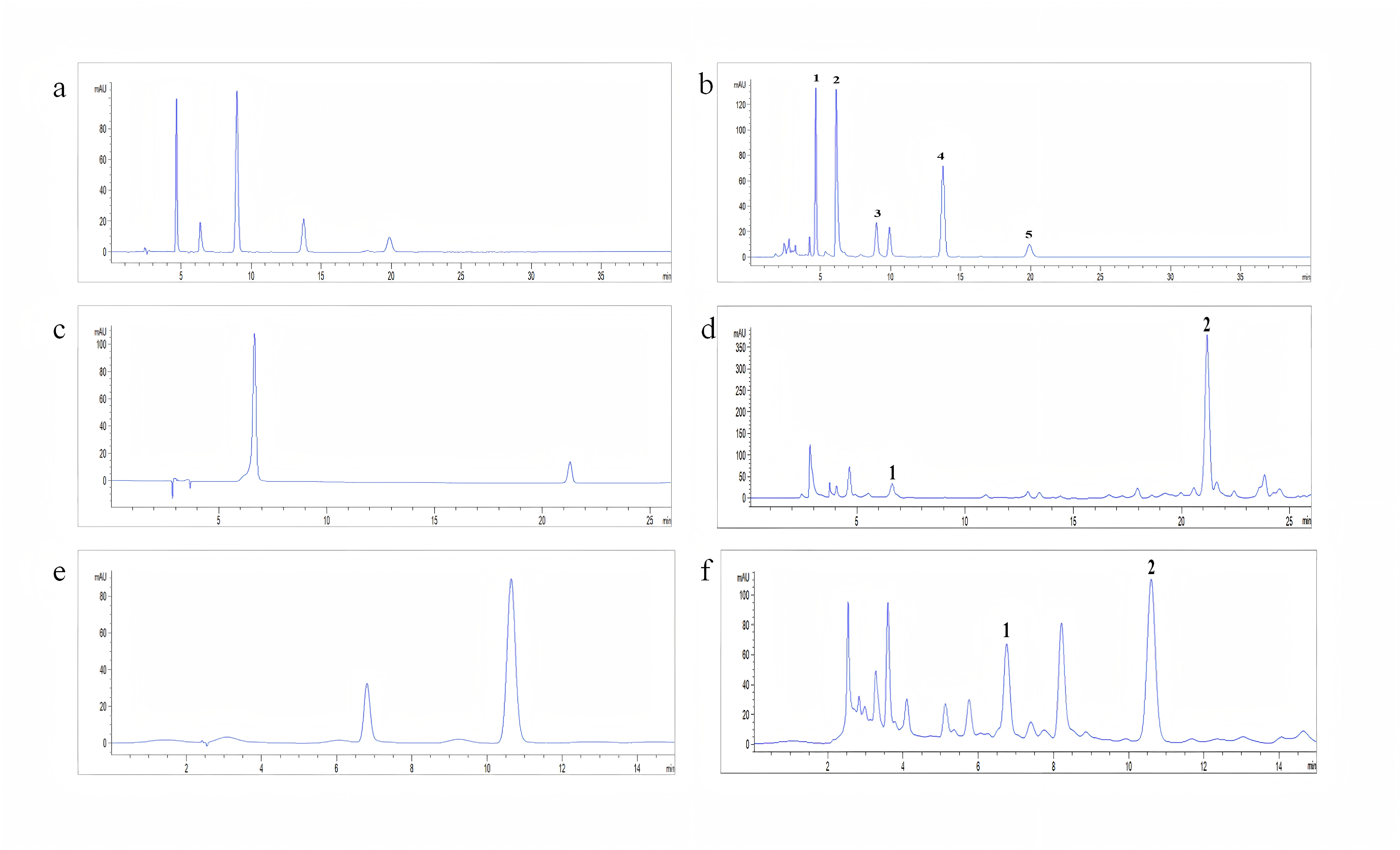

Supplement: Supplementary file 2 [file Image1.jpeg]

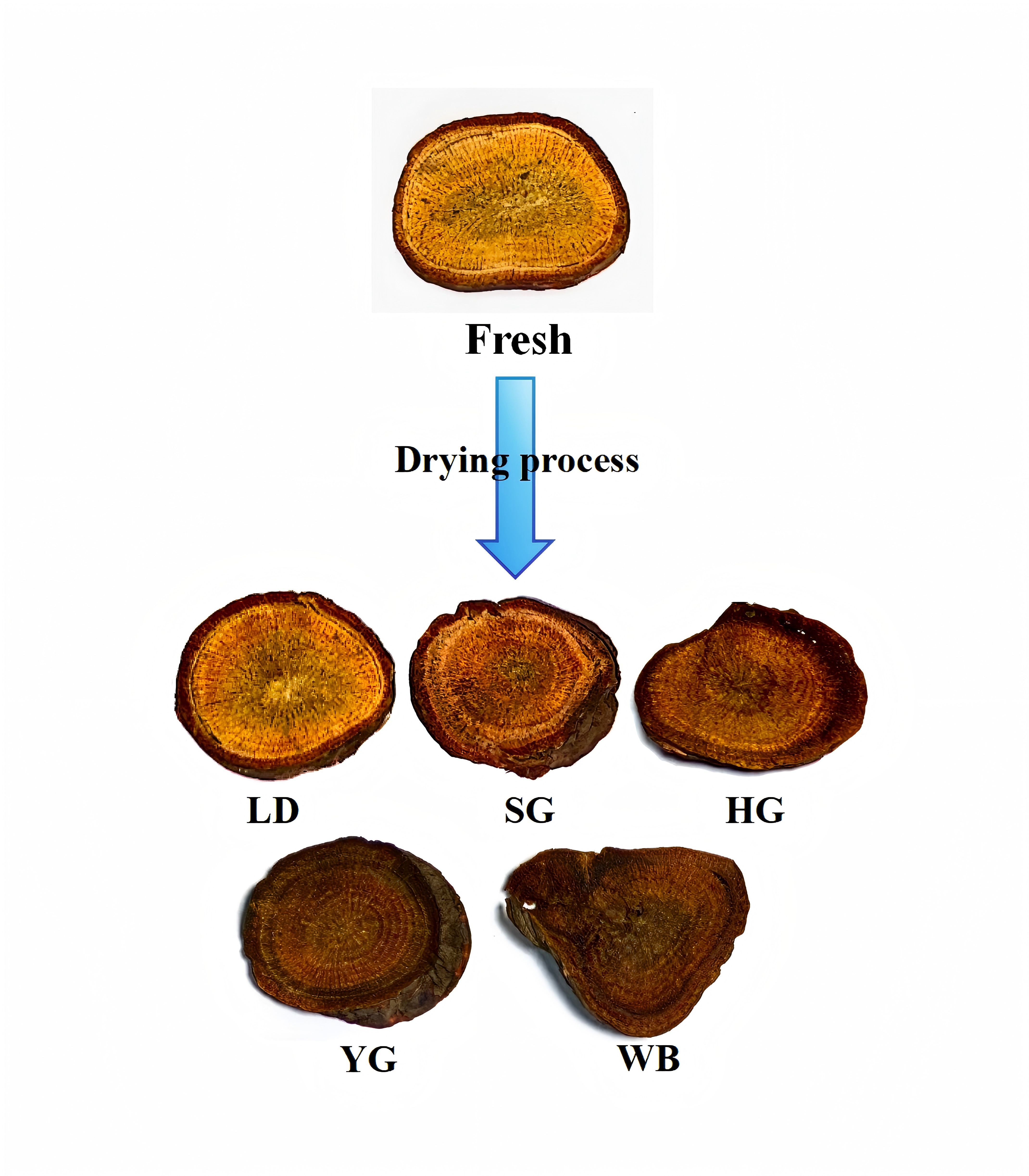

Supplement: Supplementary file 3 [file Image2.jpeg]

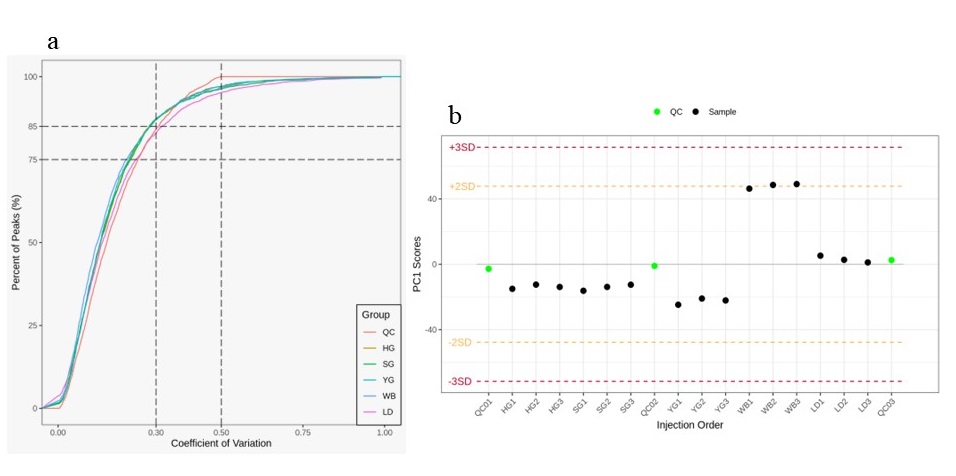

Supplement: Supplementary file 4 [file Image5.jpeg]

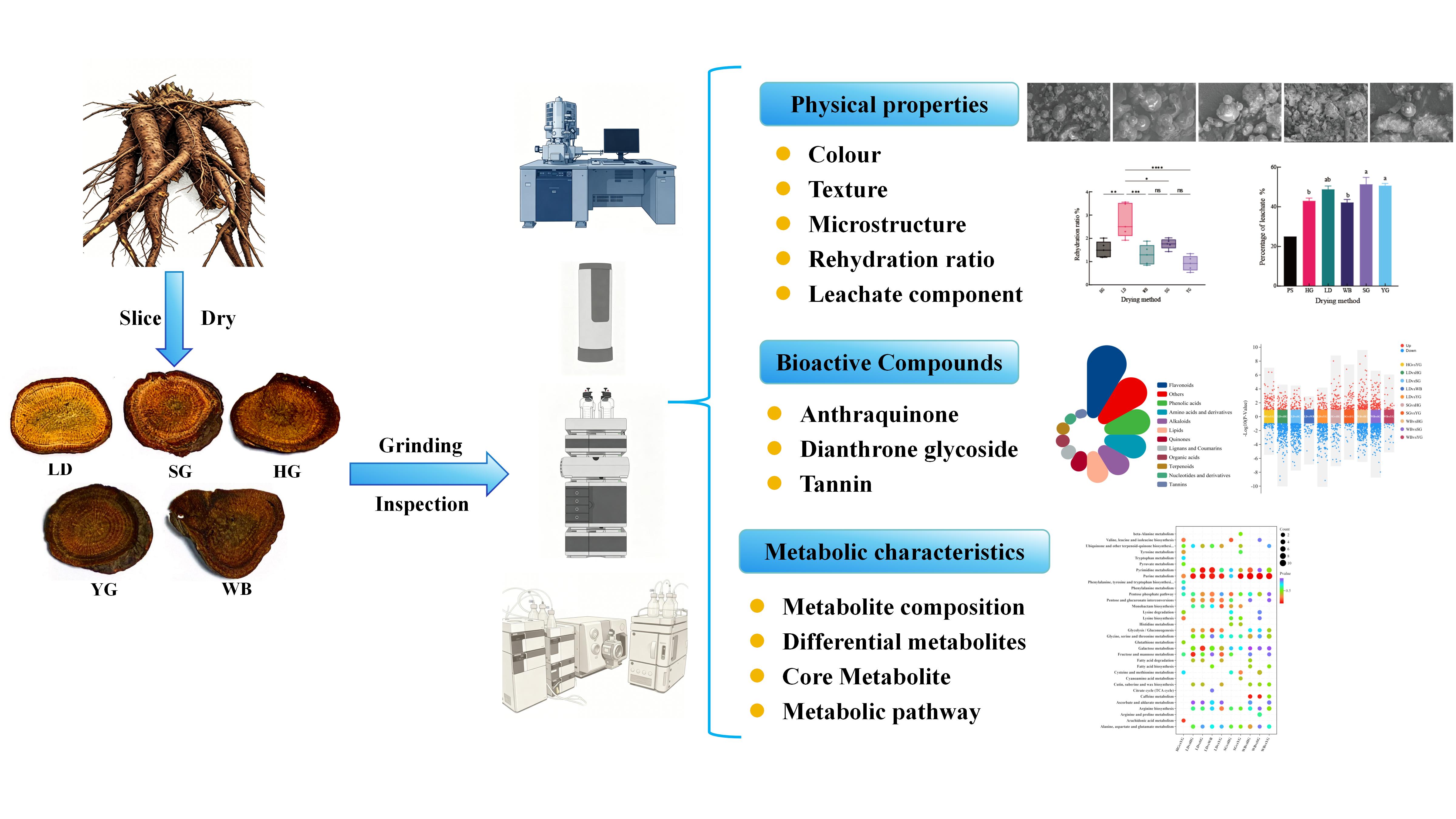

Supplement: Supplementary file 6 [file Image4.jpg]
